# Supplementary figures and images for: Overexpression of the flagellar motor protein MotB sensitizes Bacillus subtilis to aminoglycosides in a motility-independent manner
Source: PLoS One. 2024 Apr 26;19(4):e0300634. doi: 10.1371/journal.pone.0300634 (PMC11051680; doi:10.1371/journal.pone.0300634)

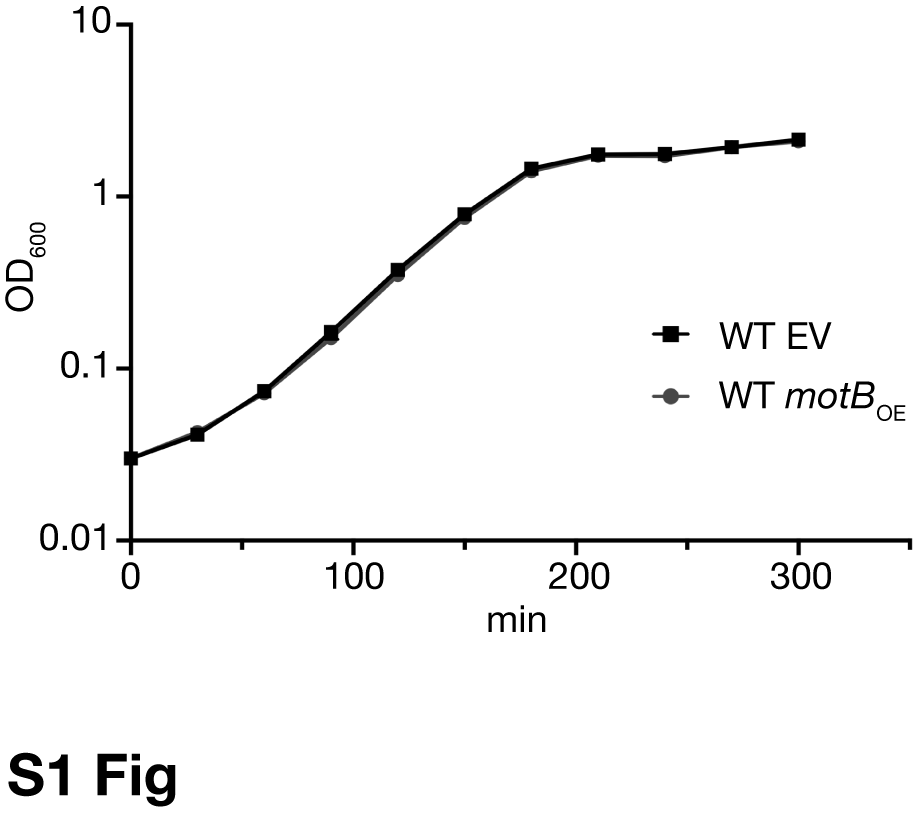

Supplement: S1 Fig — 50 μL of an OD600-adjusted overnight culture of the vector-transformed strain (WT/EV) and MotB-overexpressed strain (WT/motBOE) were aerobically cultured in 5 mL of LB containing 1 mM IPTG at 37°C. Data shown are the means ± standard errors from four independent experiments. (TIF) [file pone.0300634.s001.tif]

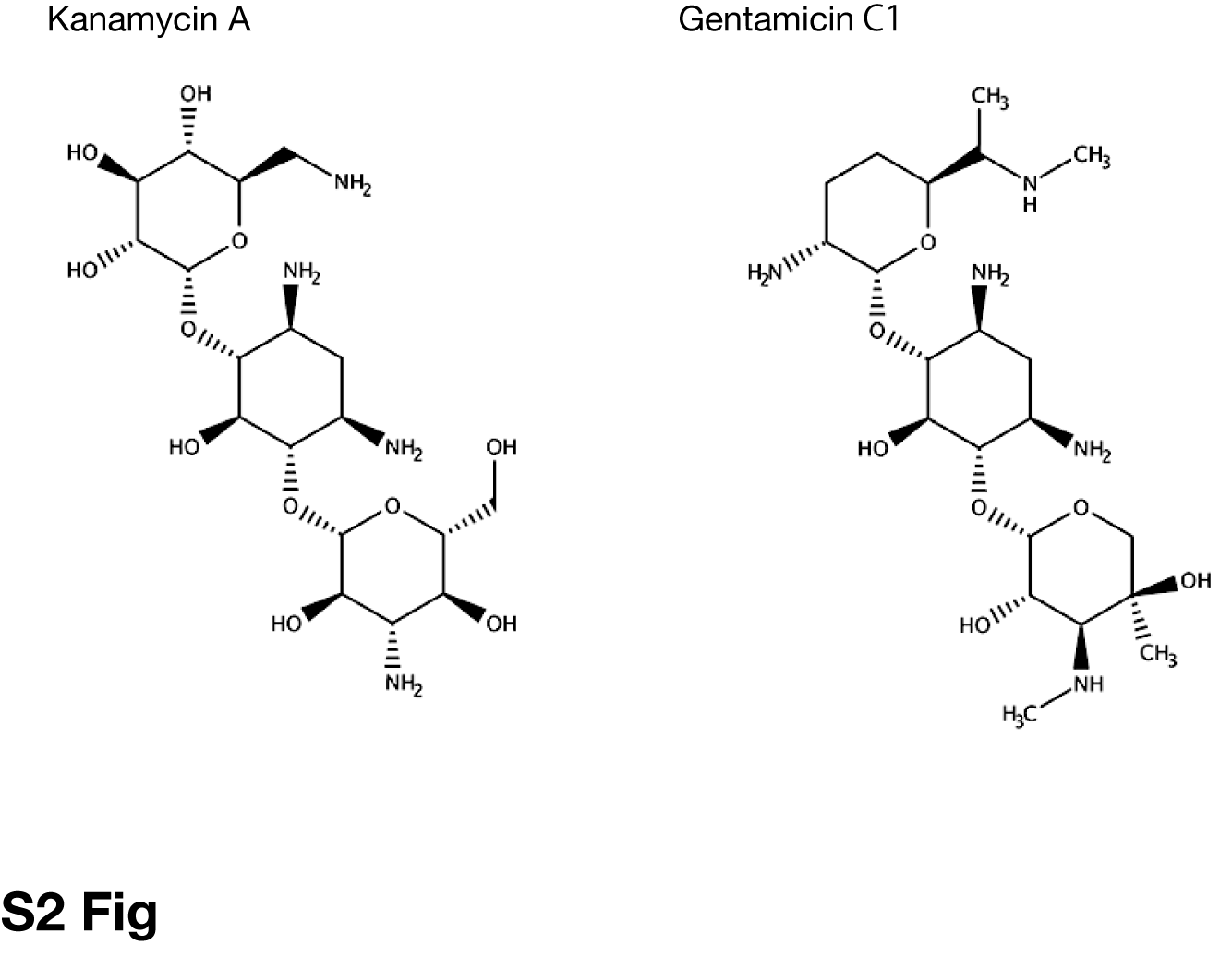

Supplement: S2 Fig — Chemical structures were constructed by Marvin Sketch of ChemAxon (16.11.21.0). (TIF) [file pone.0300634.s002.tif]

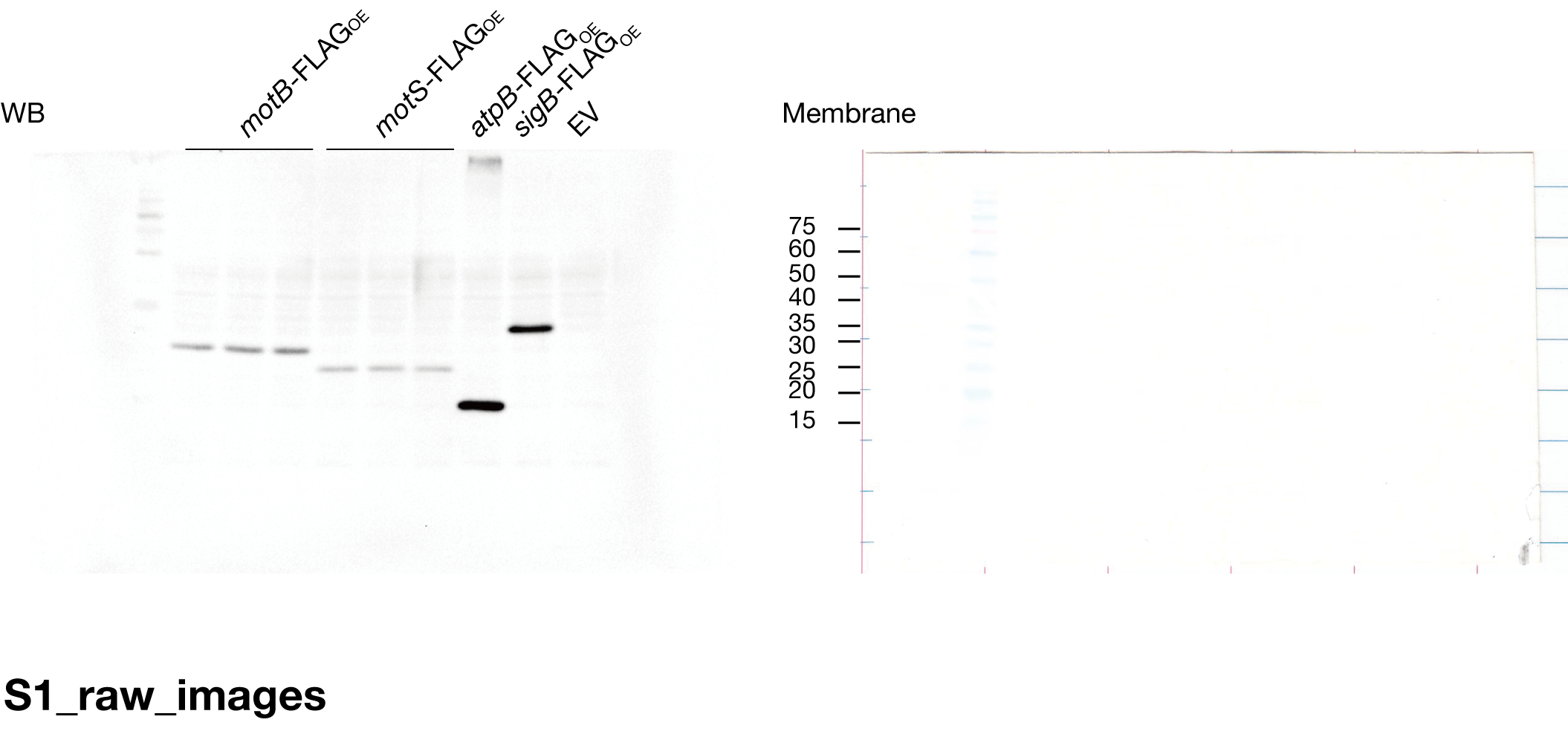

Supplement: S1 Raw images — (TIF) [file pone.0300634.s003.tif]

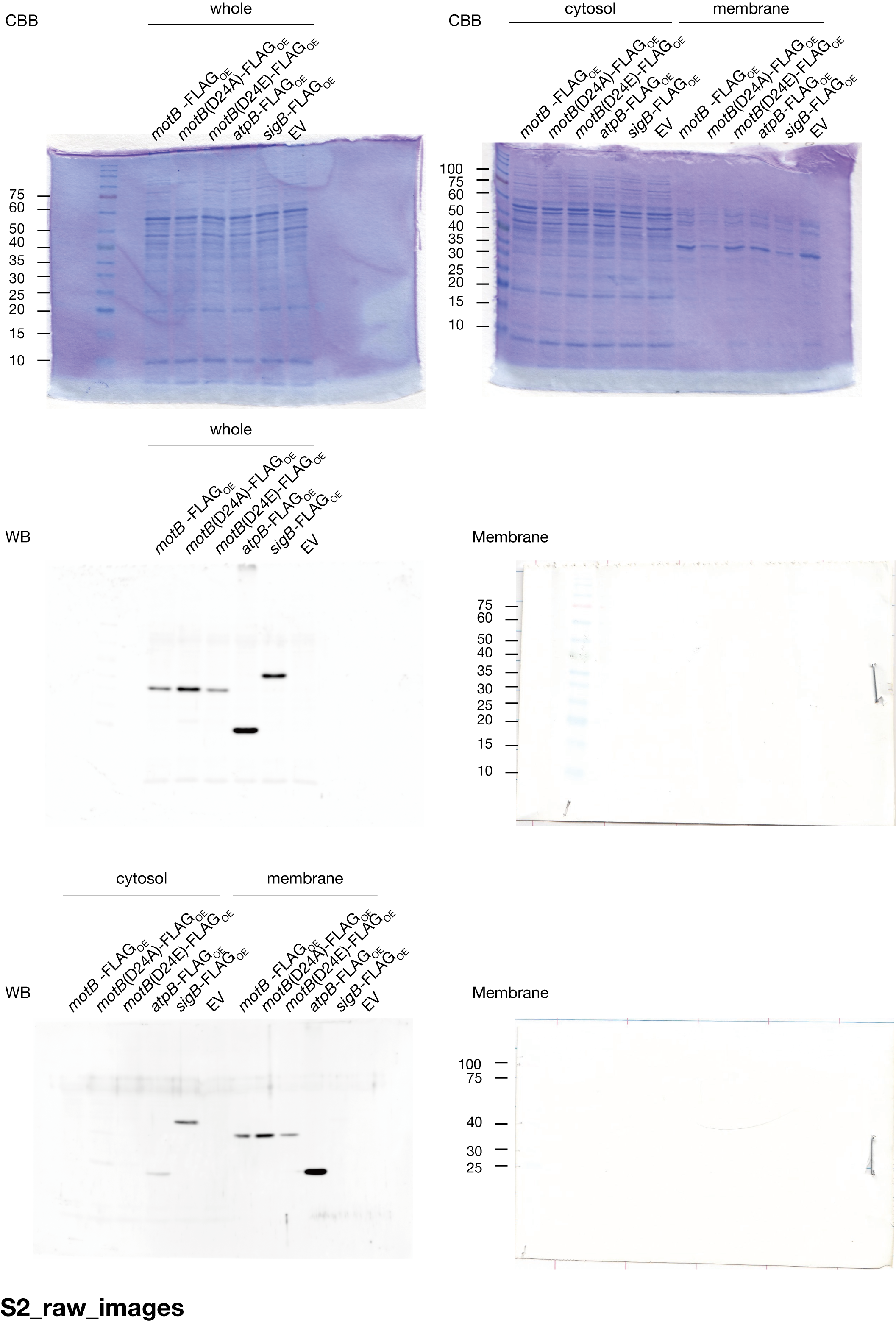

Supplement: S2 Raw images — (TIF) [file pone.0300634.s004.tif]

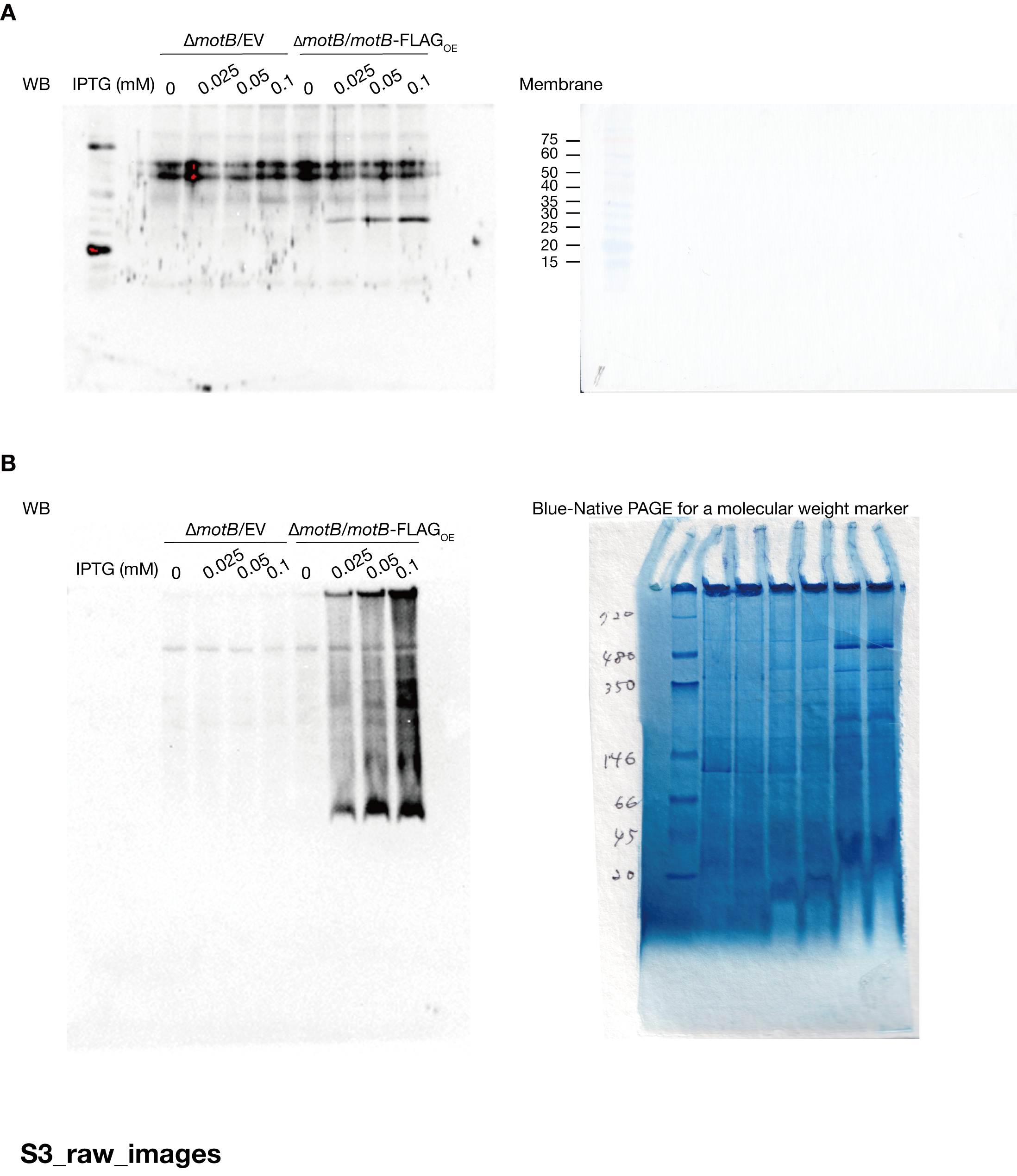

Supplement: S3 Raw images — (TIF) [file pone.0300634.s005.tif]
